# The Internet Process Addiction Test: Screening for Addictions to Processes Facilitated by the Internet

**DOI:** 10.3390/bs5030341

**Published:** 2015-07-28

**Authors:** Jason C. Northrup, Coady Lapierre, Jeffrey Kirk, Cosette Rae

**Affiliations:** 1 Department of Counseling and Human Services, St. Mary’s University, One Camino Santa Maria, San Antonio, TX 78228, USA; 2 Department of Psychology and Counseling, Texas A&M University—Central Texas, 1001 Leadership Place, Killeen, TX 76549, USA; E-Mails: lapierre@tamuct.edu (C.L.); jeff.kirk@tamuct.edu (J.K.); 3 reSTART, 16307 NE 83rd Street Suite #208, Redmond, WA 98052, USA; E-Mail: cosette.rae@restartlife.com

**Keywords:** internet addiction, internet process addiction, online video games, online social networking, online sexual activity, problematic internet use

## Abstract

The Internet Process Addiction Test (IPAT) was created to screen for potential addictive behaviors that could be facilitated by the internet. The IPAT was created with the mindset that the term “Internet addiction” is structurally problematic, as the Internet is simply the medium that one uses to access various addictive processes. The role of the internet in facilitating addictions, however, cannot be minimized. A new screening tool that effectively directed researchers and clinicians to the specific processes facilitated by the internet would therefore be useful. This study shows that the Internet Process Addiction Test (IPAT) demonstrates good validity and reliability. Four addictive processes were effectively screened for with the IPAT: Online video game playing, online social networking, online sexual activity, and web surfing. Implications for further research and limitations of the study are discussed.

## 1. Introduction

Internet addiction is characterized by extreme overuse of the Internet, resulting in negative consequences in one’s work, personal life, emotional health, or physical health [1,2,3]. It is a problem that clinicians and researchers in several countries recognize, even eliciting government intervention in some cases [4]. This phenomenon received enough attention that the Diagnostic and Statistical Manual-V (DSM-V) Development Committee recently considered (but ultimately decided to include in section 3 under conditions for further study) a variation of Internet addiction for inclusion in the DSM-V, ultimately deciding that more research was needed before formal inclusion was warranted [5]. However, some question whether or not a person can become addicted to a *medium*, such as the Internet, as opposed to the *process* that the medium facilitates [6,7,8,9,10,11]. We use the term “process” here in reference to the term *process addictions*, or “systematic behaviors mimicking the disease of addiction” [12].

The question of whether or not one becomes addicted to the Internet or a process facilitated by the Internet is an important one considering how quickly the Internet has evolved. The Internet today has myriad applications, including gaming, social networking, dating, shopping, and countless others. Problematic use of several of these applications have been the subject of several studies in recent years, providing indirect evidence for the idea that an individual becomes addicted to one or more of the many *processes* that the Internet facilitates as opposed to the Internet itself (e.g., [13,14,15,16]). Failure to recognize the distinction between addiction to the Internet as a whole and addiction to processes facilitated by the Internet could result in faulty assumptions on what the object of an individual’s addiction really is. The purpose of this study is to better differentiate what *processes* an individual might be addicted to that the Internet facilitates rather than create a test of internet addiction.

### 1.1. Internet Addiction

Many have used the term “addiction” to describe problematic Internet use for quite some time [17,18]. Recent medical research appears to support the use of this terminology in that the effects of behavioral compulsions (e.g., compulsive online video-game-playing) on dopamine pathways and other brain structures have been demonstrated to be comparable to those of chemical addictions [2,19,20]. These similar effects on the brain seem to lend credibility to the concept of *process addictions* (sometimes referred to as *behavioral addictions* or *impulse-control disorders*) in which an individual compulsively engages in a particular activity despite suffering negative consequences after repeated attempts to stop [12,21,22,23]. Examples include addictions to activities such as gambling, shopping, non-paraphilic hypersexual activities, video games and Internet use [21,22].

Young [24] was among the first to use the term “Internet addiction.” She and other researchers adapted the diagnostic criteria of pathological gambling or impulse control disorders to diagnose Internet addiction [17,18,24]. Criteria according to these definitions include preoccupation with the Internet, increasing amounts of time on the Internet, unsuccessful attempts to quit, irritability when trying to cut back, staying online longer than intended, jeopardizing significant relationships to stay online, lying to cover up Internet use, and using the Internet as an escape from problems [25]. Firm diagnostic criteria have not yet been fully agreed upon by researchers, but four components have been suggested as essential to the diagnosis: (1) excessive Internet use (especially when characterized by loss of time or neglecting basic functions); (2) withdrawal symptoms such as anger or depression when the Internet is inaccessible; (3) tolerance, exemplified by the need for increased use of the Internet to relieve negative emotional symptoms; and (4) negative consequences, such as arguments with friends or family, lying, poor school or work performance, social isolation, and fatigue [26]. Beard simply takes a holistic view of the phenomenon, stating that it occurs when “an individual’s psychological state, which includes both mental and emotional states, as well as their scholastic, occupational and social interactions, is impaired by the overuse of the medium” [27] (p. 7). 

Yet others distinguish between addiction to the Internet and addiction to the various *processes* that the Internet facilitates, arguing that the very term “Internet addiction” is misapplied, or at least should not be confused with addictions to the processes facilitated by the Internet [2,7,8,9]. Jones and Hertlein [28], for example, differentiate between the concepts of Internet addiction, sex addiction facilitated by the Internet, and Internet infidelity. Pawlikowski *et al.* [11] demonstrate noticeable differences among traits of problematic internet game players *vs.* problematic internet pornography users, supporting the idea that various types of problematic internet use be better differentiated from each other in future studies. Other examples of processes that people have compulsively used the Internet for include shopping [29], pornography [30], surfing media feeds [31], video-game-playing [32], social networking [33], and gambling [34]. We agree that the Internet is simply a medium, though the role of the medium itself should not be underestimated. The Internet has many beneficial applications, but also provides unhindered, instantaneous access to countless potentially addictive processes.

### 1.2. The Internet Addiction Test

The authors of this study decided to modify an existing instrument to better screen for process addictions. Several instruments have been created to test for Internet Addiction (or similar concepts), including the Chinese Internet Addiction Inventory (CIAI), the Compulsive Internet Use Scale (CIUS) [35], the Game Addiction Scale (GAS) [36], the Generalized Problematic Internet Use Scale (GPIUS) [37], the Internet Addiction Test (IAT) [24], the Internet Consequences Scale (ICS) [38], the Problematic Internet Use Scale (PIUS) [39], and the Problem Video Game Playing Test (PVGPT) [40], among others [41]. While all of these instruments have strong characteristics, the IAT was chosen due to its use of a cutoff point to determine problematic use, its development in an American sample (the country of origin for the sample available to the researchers), its availability in English (the language spoken by the authors), and its widespread use in the literature, The IAT [24] is a 20-item instrument that has demonstrated good reliability and validity and has been widely used to screen for Internet addiction [42,43,44]. It does not address the multiple processes facilitated by the Internet, however, but rather describes the Internet as a whole as the object of addiction. The purpose of this study is to conceptually improve upon Young’s [24] original design and create a test that examines Internet *process* addictions as opposed to simply “Internet addiction.” Such a test may provide clearer data to clinicians and researchers working with Internet process addicts.

### 1.3. Research Questions and Hypotheses

For this study, we considered the following research questions:

(1) To what extent are Internet process addictions correlated with the IAT? We hypothesize that these should be significantly positively correlated as individuals completing the IAT are probably doing so with their specific addictive process in mind while answering items. Young’s [24] test, however, does not explicitly differentiate among various processes.
(2) To what extent are specific Internet process addictions correlated with overall mental health? We hypothesize that there should be a significant negative correlation, as the presence of any addiction is usually comorbid with poor overall mental health [45]. Poor mental health would also lend support to the idea that participants with higher scores are struggling with truly *addictive* processes, and not simply a temporary problem.

## 2. Methods

### 2.1. The Internet Process Addiction Test

The instrument created for this study is the Internet *Process* Addiction Test (IPAT). It is an exploratory version of a screening instrument to see whether different types of internet-facilitated processes can be distinguished from one another. This instrument modifies and adds to Young’s [24] original design. Young’s [24] wording for the original IAT’s 20 items was altered so that instead of answering questions as they pertained to the nebulous concept of “the Internet,” participants answered similar questions *as they pertained to seven specific Internet processes*. For example, Young’s first item states, “How often do you find that you stay online longer than you intended?” [24] (p. 31). The respondent then answers the question on a 5-point Likert scale ranging between “Rarely” and “Always.” In the IPAT, the item is modified so that it reads, “How often do you find that you use the following longer than you intended?” The response area is designed so that the participant then answers the item as it applies to the following Internet processes: *Surfing* (aimlessly visiting various informational or recreational sites such as news, sports, or humor), *Online Gaming* (playing online video games), *Social Networking* (visiting social networking sites such as Facebook), *Sexual Activity* (viewing online pornography or sex chats), *Gambling* (engaging in gambling via the internet, such as online poker sites), *Cell Phone* Use (using one’s cell phone for internet access, email, games, or text messages), and *Other* (a catch-all category for areas not covered here). The same Likert-scale from the IAT is used for each process, except the additional response option of “Does Not Apply” is also provided.

The Internet can be used for countless processes, and it was difficult to choose which specific processes to include. The length of the instrument is critical to be useful to clinicians and researchers. The choice of processes to include was made in consultation with the two founding clinicians of reSTART, a residential technology addiction treatment program that has been treating individuals with problematic technology usage since 2009. One (Cosette Rae) is a MSW and the other (Hillarie Cash) is a Licensed Mental Health Counselor with a doctorate in psychology. These clinicians have worked daily with individuals trying to overcome problematic technology use. At the time of data collection, these were the only two full-time treatment providers in a residential treatment facility designed for problematic technology use in the U.S. They regularly used the IAT as part of their screening process, though at the time of data collection they were not aware of any other instruments in English in widespread use. Though they had not formally tracked specific Internet processes when first approached about this problem, they were the ones that reported that the seven most commonly seen processes for technology were the ones discussed above. Their suggestions seemed to be largely supported by the literature e.g., [11,12,13,14]. These processes were therefore included in the IPAT.

Seven questions not addressed in the IAT were added to the IPAT, as informed by Griffiths [46] and Tao *et al.* [26]. These items have the respondent rate their tendency to do the following: Minimize their use of the processes, use the processes for escapism, use other technologies to attempt to cease use of the processes, experience withdrawal symptoms (e.g., restlessness, irritability, or anxiety) when attempting to cease use of the processes, lose track of time when engaging in the processes, abandon previously enjoyed interests to engage in the processes, and engage in the processes despite harmful effects (e.g., relationship problems, missing school, missing work, or losing money).

One item from the original IAT was not adapted for inclusion in the IPAT. This item asked about respondents’ tendency to block out disturbing thoughts about life with soothing thoughts about the Internet. The authors felt that this question was too awkwardly worded when adapted, so it was removed. A few other questions were altered beyond the modifications discussed above because the questions left in their original form could unintentionally exclude some people from answering. For example, the item “How often do you neglect household chores to spend time online?” [24] (p. 31), was modified into “How often do you neglect your responsibilities to spend more time doing the following?” to keep from unintentionally excluding anyone who might not otherwise do chores. The end result of the modifications to the IAT was seven answer areas (processes) for 26 questions, totaling 182 unique items.

### 2.2. The Mental Health Inventory-5

In addition to participants completing both the IAT and the IPAT to assess concurrent validity, they also completed the Mental Health Inventory-5 (MHI-5) to examine convergent validity. The MHI-5 is a very brief (five items) instrument used to assess overall mental health in respondents [47]. It has demonstrated high validity in identifying mental health problems in respondents such as mood and anxiety disorders, despite its brevity [48]. Higher scores indicate good mental health, while lower scores indicate poorer mental health. Raw scores (5–25) are transferred to a 100-point scale. The recommended cutoff score for mood disorder is 60 or less (0.83 sensitivity, 0.78 specificity) [48]. The MHI-5 has good internal validity with a Cronbach’s alpha score of 0.74 [48].

### 2.3. Research Design

The current study was a correlational design and was used evaluate the study hypotheses regarding convergent and divergent validity with respect to comparing the newly created IPAT against the IAT and MHI-5. Additional analyses using exploratory factor analysis (principal components analysis) was employed to confirm the hypothetical constructs of the IPAT.

### 2.4. Participants

Participants were recruited via Google Ads as well as through the website of reSTART. The sample was heavy technology users averaging 7.41 (SD = 4.66, Range = 24) hours a day of non-work time where the general population uses the Internet 13 hours a week in both work and non-work time [49]. All participants were informed prior to beginning the survey that participation was voluntary, anonymous, and that they would be given feedback based on the IAT and MHI-5. Completing the survey required approximately 30 min.

Data were collected using an online assessment tool. Over the 51 week period that the survey was available, more than 1121 surveys were started. Of those submitted, 274 complete surveys were collected and 4 were removed for highly suspect data (*i.e.*, 100 year-old respondents spending 24 h online) leaving 270 complete surveys for analysis. The sample for this study consisted of 160 (59.3%) males and 110 (40.7%) females ranging in age from 19 to 79 years of age (*M* = 27.83, *SD* = 9.87). The mean age for males was 26.91 (*SD* = 10.46) and for females the average was 29.17 (*SD* = 10.52).

Of those who participated in the survey, 204 (75.6%) were self-identified as Caucasian, 18 (6.7%) Asian/Pacific Islander, 18 (6.7%) multiracial, 6 (2.2%) Black, 2 (0.7%) Native American, and 22 (8.1%) declined to identify their race. In addition, 29 (10.7%) identified their ethnicity as Hispanic.

One hundred and ninety-two (71.1%) were never married, 58 (21.5%) are currently married, 15 (5.8%) were divorced, 4 (1.5%) separated, and 1 (0.4%) was widowed.

One hundred and thirty-two (48.9%) were students, 76 (28.1%) were employed for wages, 22 (8.1%) were self-employed, 19 (7.0%) were out of work but looking, 10 (3.7%) were out of work not looking, 5 (1.9%) were homemakers, 4 (1.5%) were unable to work, and 2 (0.7%) were retired.

One hundred and one (37.4%) made less than $25,000 annually, 29 (10.7%) made between $25,000 and 35,000, 29 (10.7%) made between $35,000 and 50,000, 32 (11.9%) made between $75,000 and 100,000, 15 (5.6%) made between $100,000 and 125,000, 7 (2.6%) made between $125,000 and 150,000, and 12 (4.4%) made more than $150,000. Twenty-two (8.1%) declined to answer questions about their income.

Responses to the survey indicate that participants were primarily from the United States (68.1%), followed by Canada (5.9%), the United Kingdom (4.1%), Latin America (3.3%), Italy and Germany (1.9% each). Thirty-seven (13.8%) respondents indicated “other” and 3 (1.1%) did not offer a response to the question.

## 3. Results

Statistical analyses were conducted using Statistical Package for the Social Sciences (SPSS) 21.0 to evaluate correlations between the IAT, the IPAT, and the MHI5, investigating the validity, reliability and utility of the IPAT in relation to the other instruments.

Scores on the IAT ranged from 0–98 with a mean score of 49 and a standard deviation of 19.54. A zero order correlation was conducted between the MHI-5 and the IAT (*r* = −0.474, *p* <0.001). Subscales of the IPAT were created by summing the scores for individual survey items. Initially, this process included seven subscales: Surfing, Online Gaming, Social Networking, Cell Phone, Gambling, Sex, and Other. Participants’ responses to most IPAT subscales after controlling for demographic variables (gender, age, race, ethnicity, marital status, education level, employment, and income) were significantly correlated with their responses to the IAT as well as the MHI-5 (Table 1).

**Table 1:** Partial correlations for IAT, MHI5, and the Four IPAT Subscales *.

Measure	1	2	3	4	5	6
1. MHI 5						
2. IAT	−0.49 **					
3. Surfing	−0.47 **	0.79 **				
4. Video gaming	−0.26 **	0.43 **	0.36 **			
5. Social networking	−0.21 **	0.57 **	0.53 **	0.26 **		
6. Sex/pornography	−0.23 **	0.32 **	0.33 **	0.33 **	0.45 **	


Note: * Controlling for gender, age, race, ethnicity, marital status, education level, employment, and income; ** Coefficients significant at *p* <0.001 (2-tailed).

All IPAT subscales correlated strongly with the IAT except for Gambling. Of the remaining statistically significant correlations, the Surfing subscale correlated strongest with the IAT, *r* (259) = 0.79, *p* <0.001, while the weakest correlation was with the Sex subscale, *r* (259) = 0.32, *p* <0.001. Three of the IPAT subscales were not significantly correlated with the MHI-5, including the Gambling, Cell Phone, and Other subscales. Of the remaining statistically significant correlations, the Surfing subscale correlated strongest with the MHI-5, *r* (259) = −0.47, *p* <0.001, while the weakest correlation was with the Social Networking subscale, *r* (259) = −0.21, *p* = 0.001. After reviewing these preliminary data, the researchers decided to remove the Cell Phone, Gambling, and Other subscales due to the lack of correlation with the IAT and/or the MHI-5.

Additionally, exploratory factor analysis was conducted using principle components analysis (PCA) on the IPAT to investigate the hypothetical structure of the instrument. Using a scree-plot with eigenvalues set at 1.0, 12 components (factors) were generated. The components were then rotated using Promax and after reviewing the scree-plot it was decided to include only those items in the output with eigenvalues greater than 3.0. The resulting analysis revealed four components accounting for 78% of the variance. Factor 1 (26 items) accounted for 58.11% of the variance and measures video game addiction. Factor 2 (31 items) accounted for 10.19% of the variance and measures social networking addiction. Factor 3 (26 items) accounted for 5.95% of the variance and measures online sexual addiction. Factor 4 (15 items) accounted for 3.73% of the variance and measures internet surfing addiction. Internal consistency for each of the four subscales were measured using Cronbach’s alpha and values for each of the four subscales were 0.97 (surfing) and 0.98 (video gaming, social networking, and sex/pornography) indicating an acceptable range of reliability for the instrument. Additionally full-scale reliability was high with a value of 0.99. When compared to the IAT and the MHI-5, the IPAT demonstrated good concurrent validity with correlations ranging from 0.31–0.78 (*n* = 269, *p* <0.001) for the IAT and −0.19 to −0.46 (*n* = 269, *p* < 0.002) for the MHI-5.

## 4. Discussion

The correlations between the final IPAT subscales (Surfing, Online Gaming, Social Networking, and Sex) indicate that the IPAT has good concurrent validity. The lack of correlation between the Gambling subscale and the IAT could indicate that for gambling addicts, gambling is not as dependent on the Internet as some of the other processes. The Internet could simply be one of several methods employed to gamble.

The correlations between the final IPAT subscales and the MHI-5 indicate good convergent validity; individuals with Internet process addictions also suffer from poor overall mental health. The lack of correlation between the Gambling subscale and the MHI-5 was somewhat of a surprise, as this appears to contradict previous research that demonstrates how Internet gamblers are more likely to develop problems [50]. Coupled with the lack of correlation between the Gambling subscale and the IAT, this could indicate an inherent flaw within the Gambling subscale. In addition, the lack of correlation between the Cell Phone and Other subscales with the MHI-5 could indicate problems with the design of those constructs, as the cell phone could be seen as simply another medium and “Other” intentionally lacks specificity. These poor correlations could also indicate that individuals with those particular process addictions are not necessarily in poor mental health. These results could also simply be byproducts of a statistical analysis involving relatively low number of participants suffering from these particular process addictions in comparison to the other types of Internet process addictions measured here. In any case, these findings warrant further study.

The results of this study provide support to a growing body of work that distinguishes between several specific Internet addictions as opposed to a generalized addiction to the Internet [6,7,8,9,10,11] and also support the legitimacy of studies that have examined specific addictive processes facilitated by the Internet as opposed to the Internet as a whole [13,14,15]. These results suggest by differentiating among different processes of addiction that what is usually referred to as “Internet addiction” is really a term that could refer to any number of constructs, each of which may be require different avenues of treatment. Those that suffer from compulsive online social networking, for example, may have different treatment needs that those that suffer from online gaming addiction; yet without more accurate terminology, both may be referred to as “Internet addicts.” In addition, these results provide support for more specialized diagnostic tools that focus on specific processes such as those that focus on problematic video game playing [37,39,40]. Future instruments may prove more useful if they focus on specific processes instead of trying to focus on a broad concept like “Internet addiction”. An instrument such as a scaled-down version of the IPAT could screen for multiple processes at the same time and perhaps shed light on problems that a more generalized tool such as the IAT would not necessarily find on its own. A tool that is able to screen for multiple processes simultaneously could be useful to treatment providers who may encounter clients seeking help for one type of addictive process, not realizing that there are other processes that are potentially problematic as well.

The methodology employed does have limitations. The relatively small sample was largely White and living in the United States. The recruitment procedure resulted in a sample of convenience, which limits the generalizability of the findings. Also, future studies may consider undertaking a more formal procedure in deciding which processes to include, such as tracking patterns in a treatment setting, in order to improve the validity of the study. In addition, the large number of IPAT items (182) combined with the relatively small sample size prohibited the use of a confirmatory factor analysis to verify theoretical constructs within the IPAT. A high dropout rate contributed to this small sample size, potentially due to the large number of items. Also, as the IPAT was developed from items in the IAT and both instruments were employed, there may have been some order effect from answering similar items. The length of the various combined instruments (245 items in total) also contributed to a number of participants who discontinued the survey before completion. As with most survey approaches, the participants were self-selected and self-reported their behaviors. As there was no external evaluation it was not possible to determine clinically-based cutoff points to determine problematic levels of addiction. In addition, while the study was open to anyone, this sample was likely comprised in large part of individuals suffering from an Internet process addiction. Previous research has not focused on clearly identifying degrees of Internet process use, abuse, or addiction, but this might be accomplished by creating cutoff points at one and two standard deviations above IPAT subscale means taken from a random sample.

## 5. Conclusions

Despite these limitations, the authors are encouraged by these initial indications of validity for the IPAT. Future studies with the IPAT would benefit from confirming theoretical constructs within the IPAT. This would require recruiting larger samples and/or reducing the number of items to encourage higher completion rates. Also, future studies could attempt to compare the predictive power of the IAT and IPAT for the different processes that they claim to measure. Future studies should also attempt to determine pathological levels of Internet process addictions with a shorter instrument that might someday replace the IAT as a screening tool.
